# A new SNP genotyping technology Target SNP-seq and its application in genetic analysis of cucumber varieties

**DOI:** 10.1038/s41598-020-62518-6

**Published:** 2020-03-27

**Authors:** Jian Zhang, Jingjing Yang, Like Zhang, Jiang Luo, Hong Zhao, Jianan Zhang, Changlong Wen

**Affiliations:** 1 Beijing Vegetable Research Center, Beijing Academy of Agricultural and Forestry Sciences, National Engineering Research Center for Vegetables, Beijing, 100097 China; 2 Beijing Key Laboratory of Vegetable Germplasms Improvement, Beijing, 100097 China; 3 National Agricultural Technology Extension and Service Center, Ministry of Agriculture and Rural Affairs, Beijing, China; 4 Molbreeding Biotechnology Company, Shijiazhuang, 050000 China

**Keywords:** High-throughput screening, Plant breeding, Plant breeding

## Abstract

To facilitate the utility of SNP-based genotyping, we developed a new method called target SNP-seq which combines the advantages of multiplex PCR amplification and high throughput sequencing. Compared with KASP, Microarrays, GBS and other SNP genotyping methods, target SNP-seq is flexible both in SNPs and samples, yields high accuracy, especially when genotyping genome wide perfect SNPs with high polymorphism and conserved flanking sequences, and is cost-effective, requiring 3 days and $7 for per DNA sample to genotype hundreds of SNP loci. The present study established a DNA fingerprint of 261 cucumber varieties by target SNP-seq with 163 perfect SNPs from 4,612,350 SNPs based on 182 cucumber resequencing datasets. Four distinct subpopulations were found in 261 Chinese cucumber varieties: the north China type, the south China type, the Europe type, and the Xishuangbanna type. The north China type and Xishuangbanna type harbored lower genetic diversity, indicating greater risk of genetic erosion in these two subpopulations. Furthermore, a core set of 24 SNPs was able to distinguish 99% of the 261 cucumber varieties. 29 core cucumber backbone varieties in China were identified. Therefore, target SNP-seq provides a new way to screen out core SNP loci from the whole genome for DNA fingerprinting of crop varieties. The high efficiency and low cost of target SNP-seq is more competitive than the current SNP genotyping methods, and it has excellent application prospects in genetic research, as well as in promoting plant breeding processes in the near future.

## Introduction

A single nucleotide polymorphism (SNP) is one-base variation in a single DNA nucleotide that occurs at a specific position in the genome^1^. Compared with simple sequence repeats (SSRs), SNPs are the most abundant and stable genetic variation in genomes^2,3^. The wide occurrence of SNPs in a gene or in a regulatory region generally act as biological markers for identified genes associated with important traits^4^. In plants, SNPs have a particularly important application in reflecting both natural genetic variability and genetic drift created by breeders during plant improvement^5,6^. Thus, SNPs are ideal markers for genetic background analysis due to their advantages in high-throughput detection and easy integration of genotyping data^7^. Currently, DNA fingerprints of germplasms or varieties based on SNP markers have been constructed in several crops, such as rice^8^, maize^7^, wheat^9^, cotton^10^, soybean^11^, eggplant^12^ and pepper^13,14^, most of which are applied with DNA microarray systems.

Several traditional SNP genotyping methods based on electrophoresis systems, such as CAPS (cleaved amplified polymorphic sequence), dCAPs (derived CAPS), and AS-PCR (allele-specific PCR), are commonly used in genetic research. It is difficult to meet the rapidly increasing need for SNP genotyping^15^. The continuous progress in high-throughput sequencing has facilitated the development of some high-throughput SNP genotyping methods, such as the Gene Chip microarray or the KASP platform (competitive allele-specific PCR)^16^. While chip-based platforms require enormous amounts of time for SNP assay designing and are only suitable for genotyping hundreds of samples with thousands of SNPs^17^. KASP system also need relatively high cost for genotyping each sample. These SNP genotyping platforms often find that some SNP loci cannot be well detected and genotyped because the flanking regions of these SNPs are not conserved or there are other hits in the genome due to the sequences homology^18–20^. This causes many false positive or false negative results in SNP genotyping. Moreover, the huge cost of one-time investment in instruments or expensive microarrays also limits the use of SNPs in most laboratories. Therefore, it is necessary to use a new technology that is cost-effective and highly accurate for genotyping middle-scale (100–2000 markers) genome-wide SNPs.

At present, next-generation sequencing (NGS) technology is widely used, and the whole-genome sequences of hundreds of species have been assembled, which has promoted large-scale re-sequencing and produced millions of SNPs for genetic research^21^. Some SNP genotyping methods combing multiplex PCR and NGS technology such as the Ion Torrent platform^22,23^, AmpSeq.^24^, GBTS^25^ and Seq-SNP in LGC (LGC group, UK) have been reported, which can genotype thousands of SNPs one time. To our best knowledge, few studies have focused on analyzing extensive variation genomes and developing perfect SNP loci which harbor conserved flanking regions and have unique PCR amplification in the genome. Here, we developed a new method called target SNP-seq which combines the advantages of high-throughput sequencing and multiplex PCR amplification by using genome-wide perfect SNPs which meet the requirements of harboring conserved flanking sequences and being captured uniquely in PCR amplification. Furthermore, this approach could gain hundreds of SNP genotypes by sequencing the SNP region with an average of 1000 times coverage in a short time and at low cost.

China is the world’s top producer and consumer of cucumber, cultivating more than 1.24 million hectares and yielding about 64.87 million tons in 2017 (http://www.fao.org)^26^. As is well known, the narrow genetic background and high risk of genetic erosion cucumber has been clearly demonstrated because of hybrid varieties deriving from limited parents and breeders pursing similar breeding goals^27,28^. The annually increasing number of commercial cucumber varieties presents a major challenge for plant variety management and protection in China. Although several DNA fingerprinting research in cucumber were reported using simple satellite repeat (SSR) technology^28–30^, it was still difficult to obtain genotypes in high throughput way. Cucumber genome was the first sequenced and well assembled species in vegetables^31^, and whole genome sequencing in various cucumber germplasms made it possible to discover perfect SSR and SNP for genotyping^32^. Our previous research analyzed the genetic diversity of 382 Chinese cucumber varieties with 122 perfect SSRs by target SSR-seq technology^33^. In this study, we applied the target SNP-seq genotyping technology with 163 perfect SNPs in establishing the DNA fingerprint of 261 cucumber varieties, and revealed the population structure and genetic features in cucumber varieties.

## Materials and Methods

### Discovery of genome-wide perfect SNPs in cucumber

The first-draft cucumber genome was assembled from the Chinese cucumber inbred line 9930^31^, and its Version 2 was used as a reference genome in the present study. A source of whole-genome sequence data from 182 cucumber accessions (Supplementary Table S1) which could represent the global genetic background of cucumber were used for SNP detection, including 115 published lines (SRA056480 in NCBI)^32^. Here, SNP sites meeting the following criteria were considered as perfect SNPs: (1) only biallelic SNPs were remained; (2) the 30 bp flanking sequence of a SNP was mapped to a unique region in cucumber 9930 genome; (3) there were no Indel, SSR, or other SNP locus in 30 bp flanking region of 182 cucumber accessions; (4) heterozygosity was no more than 0.2; (5) missing rate was less than 0.2; (6) MAF (minor allele frequency) above 0.4 to ensure the SNP polymorphism in varieties. Furthermore, some SNPs with high allele frequency (≥0.95) in a certain subgroup while low allele frequency (≤0.05) in other subgroups were also selected as perfect SNPs. Finally, all perfect SNP loci were sent to the Molbreeding Biotechnology Company (Shijiazhuang, China) for multiplex PCR primer panel design.

### Construction of target SNP-seq library

**Figure 1:**
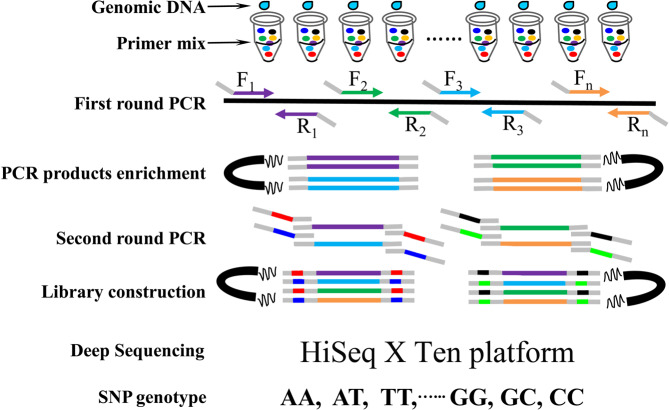
The procedure of target SNP-seq in genotyping 261 varieties with 163 perfect SNP. Library construction of target SNP-seq included two round of PCR. The first round of PCR was to capture the target SNP locus through multiplex PCR. The second round of PCR aimed to add a unique barcode adaptor for each DNA sample. Finally, the constructed library was sequenced on Highseq X platform.

The library construction of target SNP-seq consisted of two rounds of PCR (Fig. 1).The first round of PCR was to capture the target SNP locus and amplify 200–280 bp sequence in DNA samples through multiplex PCR. The second round of PCR aimed to distinguish each DNA sample by adding a unique barcode adaptor. The first round of PCR was performed in a total volume of 30 µL containing 50 ng DNA, 8 µL SNP primer mix (0.2µmol/L), and 10 µL 3 M enzymes (GenoPlexs 3 × M enzyme, MolBreeding Biotechnology Co. Ltd, http://www.molbreeding.com/). 3 M enzyme is a mixture of three kinds of enzymes including two mutant Taq enzyme and one high-fidelity thermo stable DNA polymerase. Two units (2U) of enzyme were used per reaction. The thermal cycling regime was as follows: 95 °C for 5 min, followed by 17 cycles (95 °C for 30 s, 60 °C for 4 min) and the final extension at 72 °C for 4 min. Then, the PCR products were collected by magnetic bead suspension and purified by 80% (V/V) ethanol. For the second round of PCR, the 30 µL PCR mixture consisted of 11µLpurified PCR product from the first round of PCR, 10 µL Taq enzyme, 18 µL pure water, and 1 µL barcode adaptors composed of: forward 5′-AATGATACGGCGACCA-CCGAGATCTACACTCTTTCCCTACACGACGCTCTTCCG-3′ and the reverse 5′-CAAGCAGAAGACGGCATACGAGATXXXXXXXXGTGACTGGAGTTCCTTGGCACCCGAGA-3′ (the underlined eight-base sequences indicate barcodes). PCR was run at 95 °C for 3 min followed by 7 cycles of 95 °C for 15 s, 58 °C for 15 s, 72 °C for 30 s, and the final extension 72 °C for 4 min. The products from the second round of PCR were enriched by magnetic beads, and washed three times with 100 µL 80% (V/V) ethanol and 23 µL of Tris-HCl buffer (10 mM, pH8.0–8.5). Consequently, the DNA library was sequenced on an Illumina HiSeq X Ten platform in Molbreeding Biotechnology Company. We also sequenced the DNA of the Chinese cucumber inbred line 9930 as a positive control to verify the accuracy of target SNP-seq.

### Target SNP genotype calling

In the current study, sequencing reads of 261 varieties were aligned to cucumber reference genome (9930 V2) by using BWA to determine the physical location of each target SNP amplicon^34^. SNP genotypes were generated by GATK^35^. The maximal numbers reads of SNP allele was taken as the major allele, while the other allele was taken as minor allele. To ensure the accuracy of SNP genotypes, we also filtered those sequencing reads with major allelic depth less than 20 in a variety. For heterozygous varieties, a ratio of major and minor alleles under 0.7 was treated as a heterozygous genotype. Finally, specific SNP variant bases for each variety were identified by analyzing the in-house barcodes assigned raw sequence reads.

### Plant materials and DNA extraction

A total of 261 cucumber varieties were selected in this study to evaluate the novel technique of target SNP-seq in variety identification. The 261 varieties represented all types and main cucumber varieties planted in China, including 111 commercial hybrid varieties from the Chinese seed market, 67 varieties from breeders’ collections in BVRC, 64 varieties from the Chinese government department, and 19 local landraces from Xishuangbanna of Yunnan Province in China (Supplementary Table S2). Previous studies showed that the genetic purity of hybrid seeds is prone to contamination due to the occurrence of out-crossing with foreign or self-pollinated and physical admixtures^36^. In order to increase the genotype accuracy of each cucumber variety, newly expanded young leaves grown from 30 seeds were used for DNA extraction. Total genomic DNA was isolated from freeze-dried tissue stored in 96-well plates using a plant DNA magnetic bead kit (LGC, England) according to the manufacturer’s protocol. The concentration of all extracted DNA was quantified using a Qubit2.0 fluorometer with a Quant-iT dsDNA HS Assay kit (Thermo Fisher Scientific) before targeted-SNP sequencing.

### Data analysis

#### Genetic diversity analysis of target SNPs

Various genetic diversity parameters were calculated based on the SNP genotypic data, such as the MAF (minor allele frequency), Ho (observed heterozygosity), PIC (polymorphic information content)^37^, D (Simpson’s Index of Diversity)^38^, and F (inbreeding coefficient). All these calculations were performed in Excel2016 using the following formulae:

$$\begin{array}{rcl}{\rm{PIC}} & = & 1-\mathop{\sum }\limits_{i=1}^{n}{P}_{i}^{2}-\mathop{\sum }\limits_{i=1}^{n-1}\mathop{\sum }\limits_{j=i+1}^{n}2{P}_{i}^{2}{P}_{j}^{2},\\ {\rm{D}} & = & 1-\mathop{\sum }\limits_{i=1}^{n}{P}_{i}^{2},\\ {\rm{F}} & = & 1-\frac{{H}_{o}}{He},\end{array}$$

where *P*_*i*_ and *P*_*j*_ are the frequencies of two SNP alleles among all measured varieties, n is the number of samples.

#### Population structure analysis of 261 cucumber varieties

The genetic structures of 261 cucumber varieties were analyzed by the model-based program STRUCTURE V2.3^39^. In order to determine population number (K), three parallel runs were performed for each simulated value of K ranging from 2 to 10 with the following parameters: MCMC (Markov chain Monte Carlo) replicas run for 10,000 iterations and 100,000 generations of burn-in for each K. The optimal K value depended on ΔK, using the formula defined by Evanno *et al*.^40^. To verify the optimal number of clusters, principal component analysis (PCA) was performed in the respective R package. The neighbor-joining (NJ) tree generated from MEGA7 was used to analyze the genetic relationship among 261 varieties based on the genetic distance in poppr R package. Additionally, we measured the genetic differentiation between pairs of subpopulations with an analysis of molecular variance (AMOVA) and computed the pairwise *Fst* in the poppr R package.

#### Selecting core SNP loci for cucumber variety identification

To screen out a minimal number of SNPs for distinguishing the maximal number of cucumber varieties, we used a script in Perl based on analyzing the genetic diversity of each SNP site, which was successfully used in selecting core SSR for cucumber identification^33^ and core SNP for pepper identification^13^, respectively. Detailed algorithm for core set of SNP selection was followed by MinimalMarker^41^. Finally, a core set of SNPs with the best discernibility ability was obtained and the saturation curves were plotted by the pairwise comparison of all varieties’ genotypes.

#### Selecting core cucumber varieties in the Chinese market

According to the UPOV standard for the authentic testing of crop varieties^42^, two varieties were believed to be identical when they have same genotype in a certain set of molecular markers. We set up a pairwise comparison matrix by calculating the numbers of differential SNP genotypes between each pair of varieties, and the missing genotype was treated as null. One variety has less differential SNP genotype with other cucumber varieties indicated that it has a closer kinship with others. The top 10 percent of varieties with close kinship in each subpopulation were considered as core varieties. Moreover, to verify that this comparison matrix was able to represent the kinship of 261 cucumber varieties, correlation analysis among three pairwise comparison matrix (differential SNP genotypes, genetic distance and the genetic similarity by NTSYSpc2.11^43^) were calculated by the SAS8.1 PROC CORR (SAS Institute, 1996).

## Results

### Target SNP-seq technology for genotyping SNPs

Target SNP-seq is a novel technique appropriate for mid-scale (100–2000) SNP genotyping (Fig. 1), which demonstrated higher efficiency and lower cost than existing methods. This new method combines the advantages of multiplexing PCR and targeted deep sequencing. Multiplexing PCR is commonly used in SNP genotyping because SNP fragments have non-overlapping sizes and can be separated by single capillary or gel. Based on high-throughput sequencing, multiplexing PCR can also be used in SNP genotyping and can amplify 2000 SNP loci using a single PCR^25,44^. In the second round of PCR, a unique barcode sequence was added for each DNA sample. Then, PCR products were sequenced with the Illumina X platform. We could obtain the SNP genotype data by analyzing the specific barcodes assigned to DNA samples from the raw sequence reads. This SNP genotyping process only needed 3 days for high-throughput sequencing and cost $7 for each variety. Thus, target SNP-seq is suitable for use in DNA fingerprinting, variety identification, genetic research, and molecular breeding. The SNP genotype of the inbred cucumber line 9930 from target SNP-seq was the same as that of the cucumber reference genome, indicating that this technique had good repeatability and high accuracy in SNP genotyping.

### Genome-wide perfect SNPs in the cucumber genome

**Figure 2:**
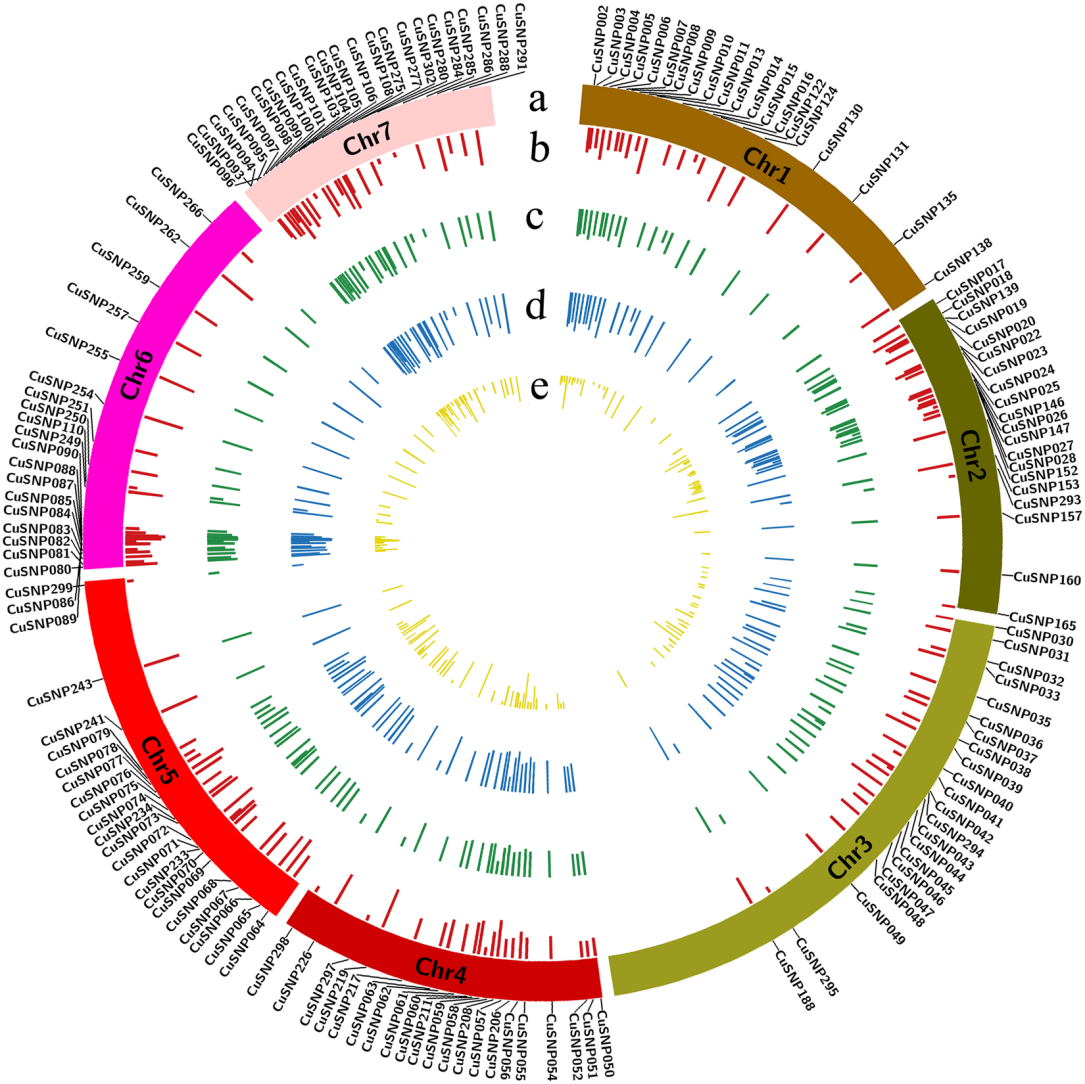
Genetic characterization of 163 SNPs in 261 cucumber varieties. (**a**) Distribution of 163 SNP loci in seven cucumber chromosomes. (**b**) Observed eterozygosity. (**c**) Genetic diversity. (**d**) Polymorphic information content (PIC) value. (**e**) Inbreeding coefficient.

In this study, we analyzed 857 Gb data from 182 cucumber accessions which had high diversity and represented all cucumber ecotypes in China^32^. A total of 4,612,350 SNPs were detected in the cucumber 9930 (V2) whole-genome sequence^31^, indicating that every 52.8 bp had one SNP. After filtering the SNP loci with MAFs above 0.4 and missing rate above 0.2, the remaining128, 434 (0.03%) SNP loci were identified. According to the objective in this study, we selected 298 perfect SNPs for multiplex PCR primer designing, which were evenly distributed on the whole genome. Ultimately, 163 SNPs composed a primer mix panel for cucumber variety identification by target SNP-seq. The number of SNP loci per chromosome ranged from 21 on chromosome 5 to 26 on chromosome 7. The average marker spacing was 1.18 Mb across the whole genome, ranging from 0.03 Mb on chromosome 6 to 6.7 Mb on chromosome 5 (Fig. 2a). The neighbor-joining (NJ) tree from 163 perfect SNPs and 128,434 SNPs had similar results in dividing 182 cucumber accessions (Supplementary Figure S1).

### Fingerprint of 261 cucumber varieties established by target SNP-seq

**Figure 3:**
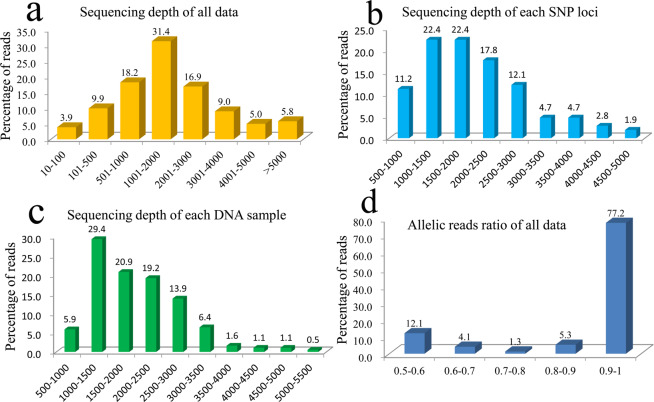
Distribution of sequencing coverage and reads ratios of target SNP-seq. (**a**) Sequence depth of all reads. (**b**) Sequence depth of 154 SNP loci. (**c**) 261 cucumber varieties. (**d**) Allelic reads ratio of homozygous and heterozygous genotype.

In present study, we genotyped 261 cucumber varieties with 163 SNP loci by target SNP-seq. A total of 84.5 million high-quality reads and 25.4 billion bases pairs were generated from 261 cucumber varieties at 163 SNP loci by the Illumina HiSeq X Ten platform. The raw data were deposited in the Genome Sequence Archive^45^(http://bigd.big.ac.cn/gsa/) under accession numbers CRA001490. The sequencing depth of 90.3% amplicons was as expected centralized in 100–5000× (50 times different) (Fig. 3a). This distribution was a comparatively ideal model in high-throughput sequencing, which ensured enough coverage for most SNP data and avoided polarized sequencing on the bulk amplicons^46^. Nine SNP loci were filtered due to low polymorphism (PIC < 0.1) and high miss rate (>0.1) in all cucumber varieties. 134 (87.0%) SNP had more than 1000× coverage and the average reads depth of the remaining 154 SNP was 2000× (Fig. 3b). The mean sequencing depth of each DNA sample was 1994×, and 246 (94.1%). varieties captured more than 1000 coverage (Fig. 3c). According to the major and minor allelic reads ratio, 16.2% of genotype data were below 0.7 while 82.5% were above 0.8 (Fig. 3d), indicating the accuracy of SNP genotype was at least above 98.7%. Based on multiplexed PCR and target deep sequencing, the target SNP-seq approach was able to provide highly accurate SNP genotypes.

The observed heterozygosity (*Ho*) of 261 varieties ranged from 0 to 0.64 while this value in154 SNPs varied from 0 to 0.44 with an average of 0.17 (Fig. 2b). Five SNPs and eight varieties exhibited higher heterozygosity (*Ho* > 0.4) (Supplementary Table S3). The PIC value ranged from 0.101 to 0.500, with an average of 0.388, while the mean value of MAF was 0.291 and varied from 0.054 to 0.496 (Fig. 2d). Interestingly, the inbreeding coefficient in three SNP loci was equal to 1, indicating that these SNPs had no heterozygous genotype in all varieties. The average genetic diversity coefficient for 261 varieties was 0.63 (Fig. 2c), indicating that 261 cucumber varieties had low diversity and high similarities in genetic background.

### Population structure analysis of cucumber varieties in China

**Figure 4:**
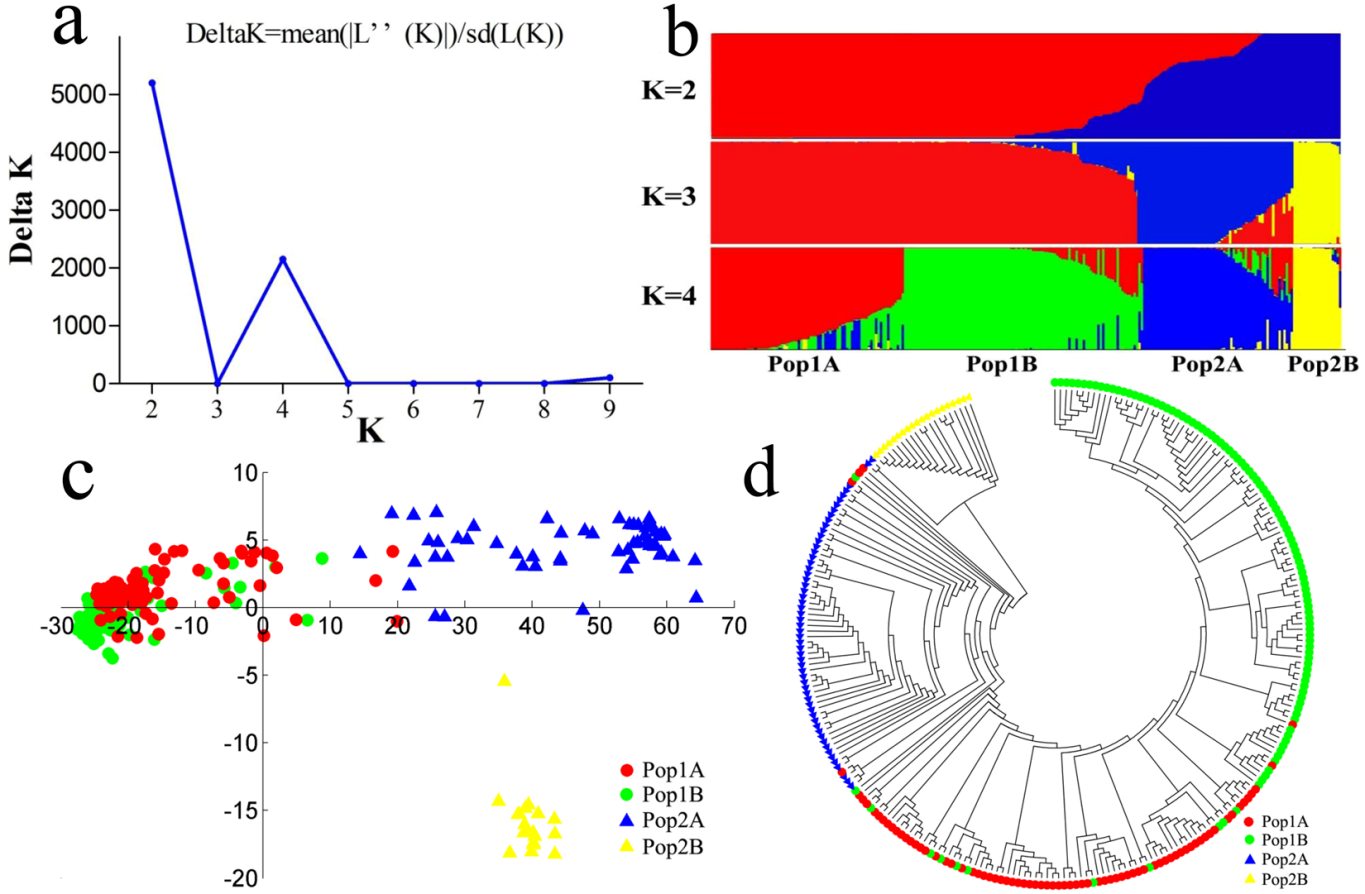
Population structure analysis in261 cucumber varieties. (**a**) ΔK plots derived from target SNP-seq genotypic data. (**b**) Population structure analysis when K = 2, 3, and 4. Pop1A is colored in red, Pop1B is colored in green, Pop2A is colored in blue, and Pop2B is colored in yellow. (**c**) PCA model score plot of 261 cucumber varieties. (**d**) neighbor-joining tree in four population structures, the color codes are the same as in (**c**).

The genetic structures of the 261 cucumber varieties were analyzed with different clusters (K from 2 to 10) using the model-based Bayesian clustering method as performed in the software STRUCTURE V2.3. The most likely number of K was 2 according to the ΔK value from the structure output (Fig. 4a).Overall, 180 varieties were assigned to subgroup 1 (Pop1) and 81 varieties were assigned to subgroup 2 (Pop2). The level of membership of 172 varieties (95%) in Pop 1 was more than >70%, while 68 varieties (84%) in Pop 2 were above this value (Fig. 4b). To detect the subpopulation of 261 cucumber varieties, we further investigated the structure changes with the increase of K value. When K = 3, Pop2 was subdivided into two clusters, one for the Europe type (Pop2A) and one for the Xishuangbanna type (Pop2B). When K = 4, the south China type (Pop1A) and north China type (Pop1B) belonging to Pop1 were assigned to independent clusters. AMOVA analysis of the four subpopulations (Pop1A, Pop1B, Pop2A, and Pop2B) indicated that 54.5% of the variation was due to differences among populations and 13.6% was due to differences within populations (Table 1). Pair wise estimates of *Fst* ranged from 0.06 to 0.58 with an average of 0.43 among four cucumber types (Supplementary Table S4), indicating that the south China type was closely related to the north China type and had strong population differentiation with the other types. The same genetic divergence among 261 cucumber varieties was also observed by PCA and neighbor-joining tree. In PCA analysis, the first axis explained 45.6% and the second axis explained 8.4% of the overall variance, respectively. The PCA plot indicated that the Europe type showed a disperse distribution while the other types presented cluster distribution (Fig. 4c). The dendrogram of 261 cucumber varieties also exhibited four distinct subgroups, and the Xishuangbanna type was first separated from other types, followed by the Europe type, south China type, and north China type (Fig. 4d).These four subpopulations were also in accordance with the geographical distribution and morphological characteristics of 261 cucumber varieties, which proved that target SNP-seq is a powerful tool for genetic analysis.

**Table 1:** Analysis of molecular variance (AMOVA) among populations and within populations.

Source of variation	Df	Sum of Square	Mean Square	Variance Components	Percentage of Variation
Between population	1	5304.8	5304.8	42.3	54.5%
Between samples Within population	2	1308.3	654.1	10.6	13.6%
Within samples	257	6352.3	24.7	24.7	31.9%
Total	260	12965.3	49.9	77.6	100.0%

### Core SNP set in cucumber variety identification

**Figure 5:**
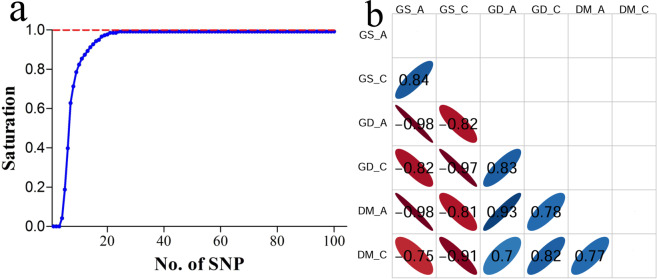
Core set of 24 SNPs represent 154 SNPs in identifying 261 cucumber varieties. (**a**) Discrimination saturation curve of 24 SNPs. (**b**) Correlation coefficients between 24 core SNPs and 154 SNPs in genetic similarity (GS), genetic distance (GD), and differential SNP markers (DM). Ellipses obliquing to right and left respectively indicate positive and negative correlations with significant *p*-values at 0.05.

The core SNP set was mainly used in variety identification and could distinguish the maximum number of varieties using the minimum number of SNP loci. A Perl script was developed to calculate the discrimination power of 154 perfect SNPs by comparing 33 930 pairwise between 261 varieties^33^. Then we acquired a core SNP set consisting of 24 SNP loci that could identify 99% of 261 Chinese commercial cucumber varieties with different SNP genotypes (Fig. 5, Supplementary Table S5). To assess the ability of the 24 core SNPs to represent 154 SNPs in variety identification, we calculated the correlation coefficients of genetic similarity, genetic distance, and differential SNP markers in 261 cucumber varieties (Fig. 5b). For all SNPs, the correlation coefficients (r) of genetic similarity with genetic distance and differential SNP markers were both –0.98. This suggests higher values of genetic similarity are associated with lower values of genetic distance and differential SNP markers. For the core SNP set, differential SNP markers had a significant negative correlation with genetic similarity (–0.97) and a significant positive correlation with genetic distance (0.82), indicating that counting differential SNP markers can be used to evaluate the genetic relationship among different varieties. The core SNP set had a significant positive correlation with all SNPs in genetic similarity (0.84), genetic distance (0.83), and differential SNP markers (0.77). Hence, the 24 core SNP loci selected in the present study were able to represent 154 SNPs in the identification of cucumber varieties from Chinese markets.

### Core cucumber varieties in the Chinese market

**Figure 6:**
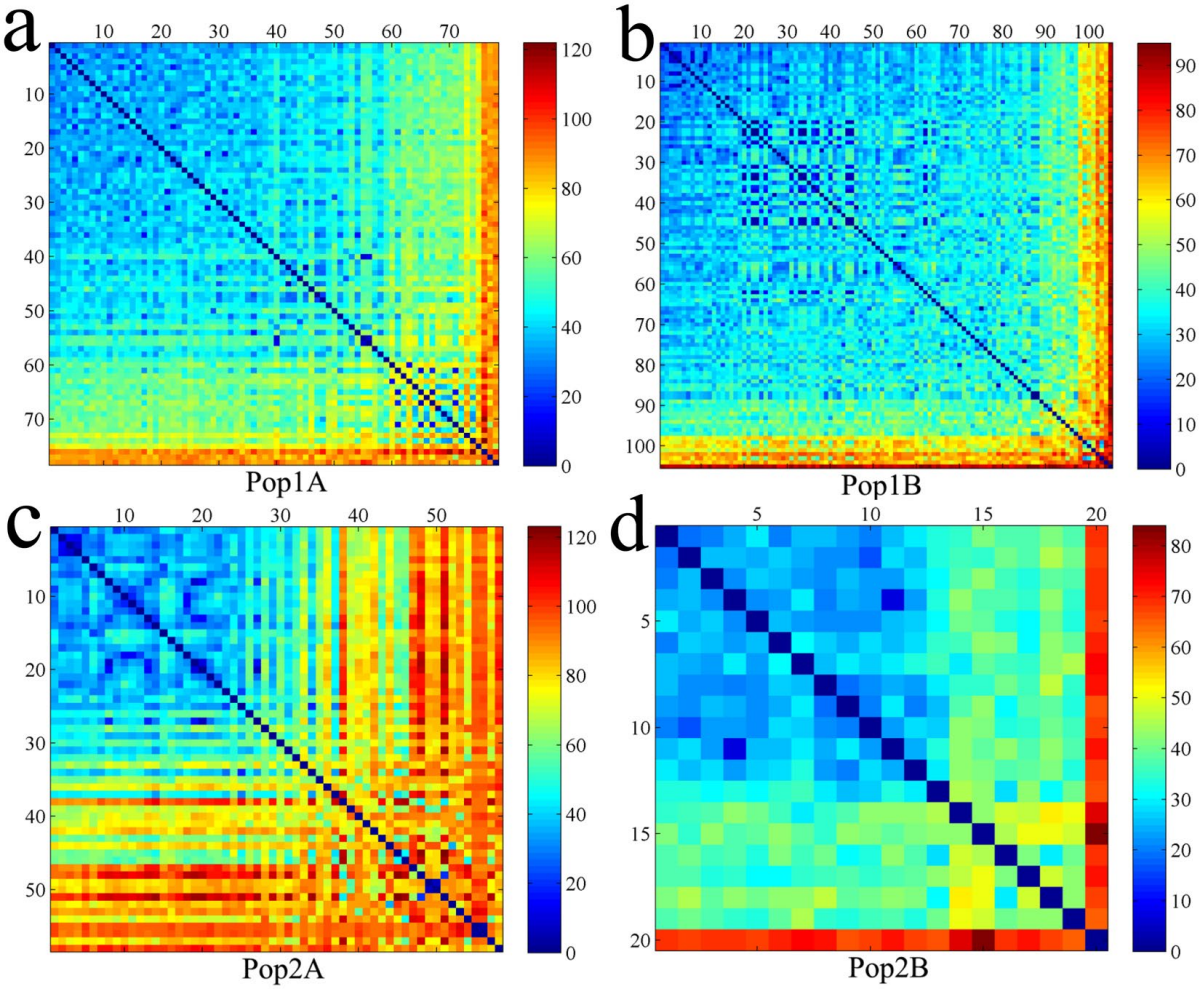
Heatmap of pairwise comparison matrix derived from differential SNP genotypes in Pop1A (**a**), Pop1B (**b**), Pop2A (**c**), and Pop2B (**d**). Blue to red indicate the increasing number of differential SNP genotypes.

As is well known, hybrid cucumber varieties in China were derived from limited germplasms and breeders pursed similar breeding goals, which result in the genetic diversity of cultivated cucumber is obviously narrow. To evaluate the genetic diversity of Chinese cucumber varieties in four subpopulations, we calculated some genetic parameters (Table 2) and developed a genetic similarity matrix by counting differential SNP genotypes between each pair of DNA samples (Fig. 6). Low PIC value and high inbreeding coefficient were found within the Xishuangbanna type (Table 2), indicating limited gene flow within this type. The average differential SNP markers in the north China type was 38.6 (Fig. 6b), while this value in the Europe type was 68.3 (Fig. 5c).The reason for this is that the north China type has a long history of variety hybridization breeding in China while the Europe type was introduced to China in recent years. Among 261 cucumber varieties, “Jinza2hao” had the highest genetic similarity with other varieties, while the foreign variety “True Lemon” had the lowest genetic similarity with others. For each subpopulation, the top 10 percent of varieties with the minimum number of differential SNP genotypes were determined as core varieties. Then, we selected a total of 29 core varieties which were the main cultivated varieties in China and had typical agronomic traits in their subpopulation (Supplementary Table S6).

**Table 2:** Genetic parameters in four sub-populations.

Subpop.	Ecotype	No. of Varieties	PIC	MAF	Ho	He	Inbreeding Coefficient	Average Differential Markers
Pop1A	South China type	78	0.241	0.171	0.171	0.327	0.477	52.4
Pop1B	North China type	105	0.175	0.122	0.161	0.442	0.637	38.6
Pop2A	Europe type	58	0.312	0.219	0.211	0.354	0.405	68.3
Pop2B	Xishuangbanna type	20	0.172	0.118	0.080	0.396	0.797	36.6

Ho: observed heterozygosity; He: expected heterozygosity; MAF: minor allele frequency; PIC: polymorphic information content.

## Discussion

### Efficient genotyping result of target SNP-seq technology

Over the past three decades, several methods for SNP genotyping have been described, and the application of SNP technology has accelerated in genetic research^47^. However, the high labor cost and low efficiency are still challenges for current SNP genotyping platforms^25,48^. Recently, some SNP genotyping methods based on NGS technology begin to spring up, such as Ampseq, GBTS and Seq-SNP, which also had some disadvantages. (1) these methods have not focus on developing perfect SNP loci through variomes big data; (2) the primers for SNP amplication need probe hybridization; (3) the sequence read depth/coverage of was less than 100× and the SNP call rate was less than 90%. In the present study, we reported the utility of target SNP-seq—a novel method for SNP genotyping and applied in genetic analysis of 261 cucumber varieties. We first designed a primer panel consisting of 163 discovered perfect SNPs in cucumber based on 182 resequencing datasets in the multiplex PCR approach of target SNP-seq. Secondly, the PCR products containing the 163 SNPs derived from 261 cucumber varieties were sequenced on the Illumina X platform. Then, the millions of reads were aligned to the cucumber reference genome (9930V2) based on strict alignment parameters, in order to obtain the accurate SNP genotypes (Fig. 3). Furthermore, in the first round of PCR, the sufficient time (4 min) for annealing was to ensure the complete amplification of different DNA and primers in multiplex PCR, which could make sure the uniformity of amplicons. Due to heterozygosis genotype was typically calculated by the ratio between major and minor raw sequencing reads, higher sequencing depth can score higher call rates and accuracy in genotyping and sequencing technologies^49^. Thus, with greater sequencing depth it’s easier to both capture the right sequences in the genome and the right position in bioinformatic analyses which could reduce the rate of false positives and false negatives. In this study, the average read depth of the selected perfect SNPs was more than 1000×, and the statistical accuracy of SNP genotyping in target SNP-seq was as high as 98.7% (Fig. 3d). Therefore, the target SNP-seq method had obvious advantages in the genotyping hundreds of SNPs compared with traditional SNP genotyping methods, and the total procedure only cost 3 days and $7 for each DNA sample (i.e., variety in this study).

### Genetic variation analysis of Chinese cucumber varieties

Cucumber is recognized to have originated in the Himalayan Mountains, and has been domesticated for nearly 3500 years^50^. According to morphological characteristics and geographical distribution, more than 3000 cucumber germplasms and the core set of 115 cucumber accessions were classified the into four types as follows: Indian group, Eurasian group, East Asian group, and Xishuangbanna group^27^. The cultivation of cucumber in China has a long history, dating back to the Han dynasty according to historic records, when the diplomat Zhang Qian brought back cucumber through the Silk Route^27^.

Although the genetic diversity of cucumber germplasm resources in China has been well investigated by molecular markers, relatively few markers or varieties were tested, and a comprehensive study of cultivated varieties in the Chinese market was lacking^27,32^. Because the assessment of genetic variation and relationship in cultivated varieties is essential for plant variety protection and breeding novel varieties to satisfy customers worldwide^51^, this study analyzed 261 dominant cucumber varieties in the Chinese market using target SNP-seq with 163 genome-wide perfect SNPs, and we found that Chinese cucumber varieties could be classified to four subgroups (i.e., south China type, north China type, Xishuangbanna type, and Europe type; Fig. 4), of which the first two types were supposed to be derived from the previously reported East Asian group^27^. The Xishuangbanna type had higher inbreeding coefficients and further genetic distance than the other three types (Table 2, Supplementary Table S4), which was consistent with the semi-wild cucumber type reported by previous studies^27,32^. Our previous study also found that the South China type and Europe type had higher genetic diversity than that in north China type and Xishuangbanna type^33^, indicating the narrow genetic background of the latter two types. The close similarity and narrow genetic diversity of cucumber varieties implies a risk of genetic erosion in the breeding process caused by narrowing the exploitation of new genes^52^. Therefore, it is urgent to increase the genetic diversity of varieties in the north China type and Xishuangbanna type by introgressing some favorable genes from natural resources into the breeding system.

### DNA fingerprint based on target perfect SNPs in cucumber

In order to protect the intellectual property of crop breeders, each new candidate variety must undergo DUS (distinctness, uniformity, and stability)^42^ testing. China is the world’s top producer and consumer of cucumber, which was challenged in DUS testing and varieties management^53^ due to the rapid increase in the number of cucumber varieties every year. It is well known that the molecular markers have advantages in identifying varieties, including their co-dominance, high reproducibility, and the fact that they are free from environmental impact, compared with the phenotypic or morphological characters by traditional field inspection^54^. The SNP technology was applied to variety identification and DNA fingerprinting because it is preferable to high-throughput genotyping, which was studied based on microarrays or other systems in several crops such as maize, wheat, etc^7,9,11^. However, the high cost for each variety and the large amount of time consumed make the wide use of this technology difficult in variety identification and management, in addition to the proportion of false positives or negatives caused by SNPs with diverse flanking sequences or non-specific capturing. It is also difficult to select a few SNP loci for variety identification from the millions of SNPs in whole genomes. The present study took the lead in the use of a set of 163 perfect SNP loci which had conserved flanking sequences and unique amplification in the cucumber genome, analyzing the 182 cucumber accessions’ resequencing datasets, and establishing an accurate DNA fingerprint for each cucumber variety using target SNP-seq (Fig. 2). Then, a core set of 24 SNP loci was calculated to distinguish 99% of 261 cucumber varieties, representing a discriminating capacity as high as 98% of the total 163 SNPs (Fig. 5a,b). Our results are of great importance for the identification of the authenticity and measurement of the purity of cucumber varieties, and for the protection of plant varieties in the future.

### Wide application prospects of target SNP-seq in the plant breeding system

Over several decades of effort, marker-assisted selection (MAS) breeding has enhanced the efficiency of breeding at a dramatic speed in many crops^55^. With the rapid development of genome sequencing and resequencing, hundreds of plant genomes have been assembled and genome-wide variomes have been studied, making it easy to construct background selection by the MAS system in breeding. Furthermore, as new powerful functional markers, thousands of SNP loci or SNP haplotypes have been thoroughly investigated in plants, making it possible to select individuals with disease resistance and specific agronomic traits at the seedling stage^6,56–59^. The increasing demands of background selection and foreground selection call for new SNP genotyping methods to screen hundreds of SNPs simultaneously at low cost of time and money. In this study, target SNP-seq was proved as a flexible, efficient, and affordable SNP genotyping technique that could see wide use in the modern breeding system, including background selection, foreground selection, pyramiding breeding, breeding by design, and variety authenticity identification and purity inspection. Consequently, target SNP-seq technology will undoubtedly see extensive application prospects in genetic research and breeding of novel varieties in the near future.

## Conclusions

In the present study, we established a DNA fingerprint of 261 cucumber varieties with 163 perfect SNPs by target SNP-seq technique. Four distinct subpopulations were found in 261 Chinese cucumber varieties: the north China type, the south China type, the Europe type, and the Xishuangbanna type. A core set of 24 SNPs was able to distinguish 99% of the 261 cucumber varieties, and 29 core cucumber backbone varieties in China were identified. Therefore, target SNP-seq has excellent application prospects in genetic research, and in promoting plant breeding processes, in the near future.

## Supplementary information

Supplementary Figures.

Supplementary Table S1.

Supplementary Table S2.

Supplementary Table S3.

Supplementary Table S4.

Supplementary Table S5.

Supplementary Table S6.
