# Author Correction: A new SNP genotyping technology Target SNP-seq and its application in genetic analysis of cucumber varieties

**DOI:** 10.1038/s41598-021-86981-x

**Published:** 2021-04-07

**Authors:** Jian Zhang, Jingjing Yang, Like Zhang, Jiang Luo, Hong Zhao, Jianan Zhang, Changlong Wen

**Affiliations:** 1 Beijing Vegetable Research Center, Beijing Academy of Agricultural and Forestry Sciences, National Engineering Research Center for Vegetables, Beijing, 100097 China; 2 Beijing Key Laboratory of Vegetable Germplasms Improvement, Beijing, 100097 China; 3 National Agricultural Technology Extension and Service Center, Ministry of Agriculture and Rural Affairs, Beijing, China; 4 Molbreeding Biotechnology Company, Shijiazhuang, 050000 China

Correction to: *Scientific Reports* 10.1038/s41598-020-62518-6, published online 27 March 2020

The original version of this Article contained an error in the Results section, under the subheading ‘Genome-wide perfect SNPs in the cucumber genome’, where

“The neighbor-joining (NJ) tree from 163 perfect SNPs and 128,434 SNPs had similar results in dividing 182 cucumber accessions (Supplementary Table S1).”

now reads:

“The neighbor-joining (NJ) tree from 163 perfect SNPs and 128,434 SNPs had similar results in dividing 182 cucumber accessions (Supplementary Figure S1).”

In addition, the Supplementary Information published with this Article contained errors. In the original Supplementary Information file, the primers in Table S5 were truncated.

These errors have now been corrected in the PDF and HTML versions of the Article and in the accompanying Supplementary Information files.

