# Supplementary material for: A new SNP genotyping technology Target SNP-seq and its application in genetic analysis of cucumber varieties
Source: Sci Rep. 2020 Mar 27;10:5623. doi: 10.1038/s41598-020-62518-6 (PMC7101363; doi:10.1038/s41598-020-62518-6)
Supplement: Supplementary file 1 — Supplementary Figures. [file 41598_2020_62518_MOESM1_ESM.docx]

**A new SNP genotyping technology Target SNP-seq and its application in genetic analysis of cucumber varieties**

Jian Zhang ^1,2*,^ Jingjing Yang ^1,2*,^ Like Zhang ^3*^, Jiang Luo ^1,2^, Hong Zhao ^1,2^, Jianan Zhang ^4^, Changlong Wen 1,2

1 Beijing Vegetable Research Center, Beijing Academy of Agricultural and Forestry Sciences, National Engineering Research Center for Vegetables, Beijing 100097, China.^2^ Beijing Key Laboratory of Vegetable Germplasms Improvement, Beijing 100097, China.^3^ National Agricultural Technology Extension and Service Center, Ministry of Agriculture and Rural Affairs, China.^4^ Molbreeding Biotechnology Company, Shijiazhuang 050000, China. *These authors contributed equally to this work. Correspondence and requests for materials should be addressed to C.-l.W. ([email: wenchanglong@nercv.org)](mailto:wenchanglong@nercv.org))


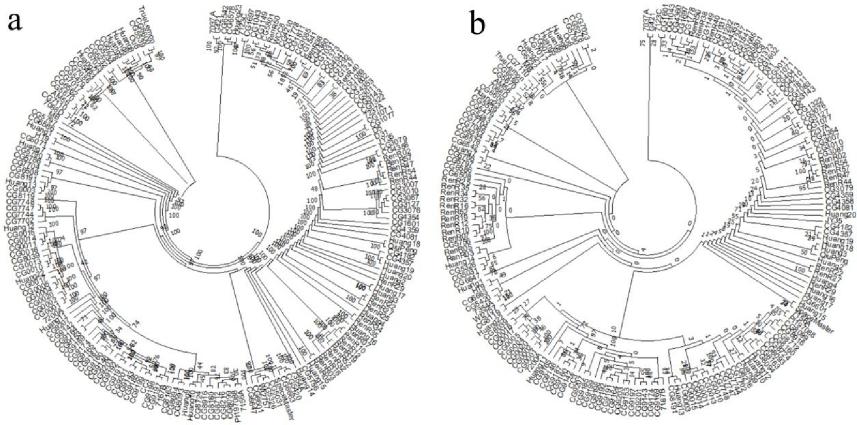


**Figure S1.** Neighbor-joining (NJ) tree of 182 cucumber accessions from 128,434 SNPs (a) and 163 perfect SNPs (b).


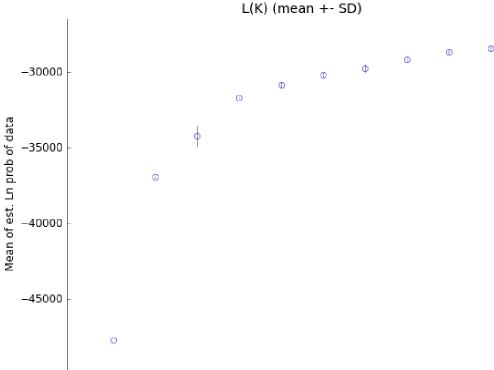


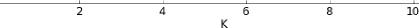


**Figure S2.** Mean and standard deviation of L(K) for each K value
